# Redox Imbalances in Ageing and Metabolic Alterations: Implications in Cancer and Cardiac Diseases. An Overview from the Working Group of Cardiotoxicity and Cardioprotection of the Italian Society of Cardiology (SIC)

**DOI:** 10.3390/antiox9070641

**Published:** 2020-07-21

**Authors:** Valentina Mercurio, Alessandra Cuomo, Christian Cadeddu Dessalvi, Martino Deidda, Daniela Di Lisi, Giuseppina Novo, Roberta Manganaro, Concetta Zito, Ciro Santoro, Pietro Ameri, Paolo Spallarossa, Eleonora Arboscello, Carlo Gabriele Tocchetti, Claudia Penna

**Affiliations:** 1 Department of Translational Medical Sciences, Federico II University, 80131 Naples, Italy; valemercurio@yahoo.com (V.M.); alebcuomo@gmail.com (A.C.); 2 Department of Medical Sciences and Public Health, University of Cagliari, 09042 Cagliari, Italy; cadedduc@unica.it (C.C.D.); martino.deidda@tiscali.it (M.D.); 3 Cardiology Unit AUOP Policlinico, Department of Health Promotion, Mother and Child Care, Internal Medicine and Medical Specialties, University of Palermo, 90127 Palermo, Italy; danydilis@hotmail.it (D.D.L.); giuseppina.novo@unipa.it (G.N.); 4 Cardiology with Coronary Intensive Care Unit, Department of Clinical and Experimental Medicine, University Hospital Policlinico “G. Martino”, University of Messina, 98124 Messina, Italy; manganaro.roberta@gmail.com (R.M.); czito@unime.it (C.Z.); 5 Department of Advanced Biomedical Sciences, Federico II University, 80131 Naples, Italy; cirohsantoro@gmail.com; 6 Cardiovascular Disease Unit, IRCCS Ospedale Policlinico San Martino, Genova, Italy—IRCCS Italian Cardiovascular Network & Department of Internal Medicine, University of Genova, 16121 Genova, Italy; pietroameri@unige.it (P.A.); paolo.spallarossa@unige.it (P.S.); eleonora.arboscello@alice.it (E.A.); 7 Interdepartmental Center of Clinical and Translational Sciences, Federico II University, 80131 Naples, Italy; 8 Department of Clinical and Biological Sciences, University of Turin, 10043 Torino, Italy

**Keywords:** cancer, cardiovascular toxicity from anticancer drugs, cardiovascular disease, ageing, metabolic syndrome

## Abstract

Metabolic syndrome (MetS) is a well established risk factor for cardiovascular (CV) diseases. In addition, several studies indicate that MetS correlates with the increased risk of cancer in adults. The mechanisms linking MetS and cancer are not fully understood. Several risk factors involved in MetS are also cancer risk factors, such as the consumption of high calorie-food or high fat intake, low fibre intake, and sedentary lifestyle. Other common aspects of both cancer and MetS are oxidative stress and inflammation. In addition, some anticancer treatments can induce cardiotoxicity, including, for instance, left ventricular (LV) dysfunction and heart failure (HF), endothelial dysfunction and hypertension. In this review, we analyse several aspects of MetS, cancer and cardiotoxicity from anticancer drugs. In particular, we focus on oxidative stress in ageing, cancer and CV diseases, and we analyse the connections among CV risk factors, cancer and cardiotoxicity from anticancer drugs.

## 1. Introduction

Metabolic syndrome (MetS) and its related conditions are serious health problems worldwide. Indeed, MetS includes several pathological manifestations such as hypertension, abdominal obesity and dyslipidaemia, hyperglycaemia and diabetes mellitus. Although MetS is strictly linked to cardiovascular diseases (CVDs), nowadays several studies indicate that MetS also correlates with the presence of an increased risk of cancer in adults [1]. The mechanisms behind this MetS–cancer link is not fully understood. Several risk factors involved in MetS are also cancer risk factors, such as hypercaloric food and/or high-fat intake, low fibre intake, and sedentary life habits. Oxidative stress is a common aspect in both cancer and MetS [2,3]. Visceral obesity is characterised by a state of low-level chronic systemic inflammation, with the creation of a pro-tumorigenic environment induced by the production of inflammatory cytokines by both adipocytes and infiltrating immune cells. Inflammation is the pabulum on which metabolic stress is superimposed [4].

Drugs used in cancer are known to induce potentially serious cardiotoxic effects (Table 1), especially in patients with CVDs and other comorbidities, including diabetes mellitus, obesity and MetS. In this review, we discuss the interplay among MetS, cancer and cardiotoxicity in men and women. In particular, we focus on oxidative stress in ageing, cancer and CVDs, and we analyse the role of hypercholesterolemia, obesity, hypertension and diabetes on cardiotoxicity from anticancer drugs.

## 2. Oxidative Stress in Ageing, Cancer and Cardiovascular Diseases

Ageing is characterised by an increase in the prevalence of several chronic and degenerative conditions, such as cancer and CVDs, with the involvement of oxidative stress and cellular senescence [11,12]. Given the close relationship between oxidative stress, inflammation, and ageing, the oxidation-inflammatory theory of ageing, or oxi-inflamm-ageing, proposes that ageing is a loss of homeostasis due to chronic oxidative stress that affects especially the regulatory systems, such as the nervous, endocrine, and immune systems. This generates an inflammatory state that leads to a vicious circle in which chronic oxidative stress and inflammation feed each other, hence enhancing age-related morbidity and mortality [13]. Indeed, CV risk factors (i.e., obesity, diabetes, hypertension, and atherosclerosis) are associated with the increased inflammation mediated by interleukin (IL-) IL-1α, IL-6, IL-8, and increased cellular senescence [14,15], while reactive oxygen species (ROS) mediate the induction of epithelial to mesenchymal transition, inducing tumour cancer metastasis [2,16]. Changes in the epidemiology of cancer and CVDs have also made the co-occurrence of these two diseases more likely, as exemplified by the concomitance of cancer and heart failure (HF). Thanks to advances in pharmacological and device therapies, along with a holistic approach provided by multidisciplinary teams, CV death, sudden cardiac death in particular [17,18,19], has been reduced among patients with HF. However, this has led to an increased burden of co-morbidities, including cancer [20]. On the opposite, the parallel amelioration of oncological management and treatments has significantly decreased the mortality linked to several cancers, while concomitantly increasing the comorbidity burden of oncological elderly patients. Several studies demonstrated that CV disease is the most frequent non-cancer cause of death in cancer patients [5], and an increased risk of incident HF has been reported amongst patients diagnosed with cancer. This is mainly due to the CV toxicity of anti-neoplastic drugs and/or radiation therapy [6] but novel studies suggest that this association extends beyond the effects of cancer therapy. In fact, cancer and HF share many common risk factors that may predispose to both conditions.

Importantly, cancer may promote HF development, and HF per se may also induce tumour progression [21]. Beyond this, the two conditions appear to share common systemic pathogenic pathways and mechanisms that may in part explain their association [22]. Hence, the connections between HF (and other CVDs) and cancer have emerged as a new discipline that encourages collaborations between oncologists and cardiologists at clinical and research levels, and thereby aims at optimising the management of the individuals affected by these diseases. In most clinical studies, both conditions are mutually excluded. When assessed, however, cancer accounted for a substantial proportion of non-CV deaths in HF trials [20,23]. There is also the need to establish a universal set of biomarkers, including biochemical and imaging modalities that should be integrated in such studies [7].

**Figure 1:**
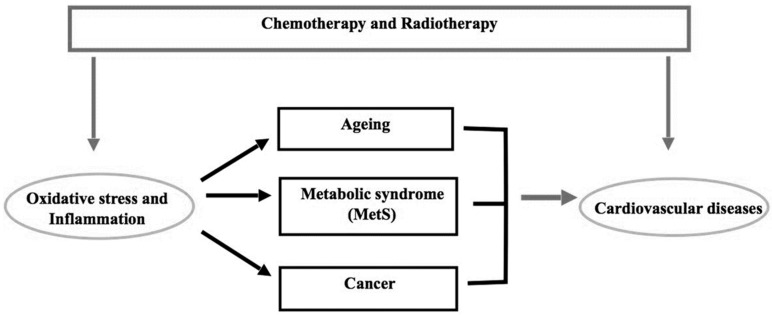
Metabolic syndrome and cardiotoxicity.

**Figure 2:**
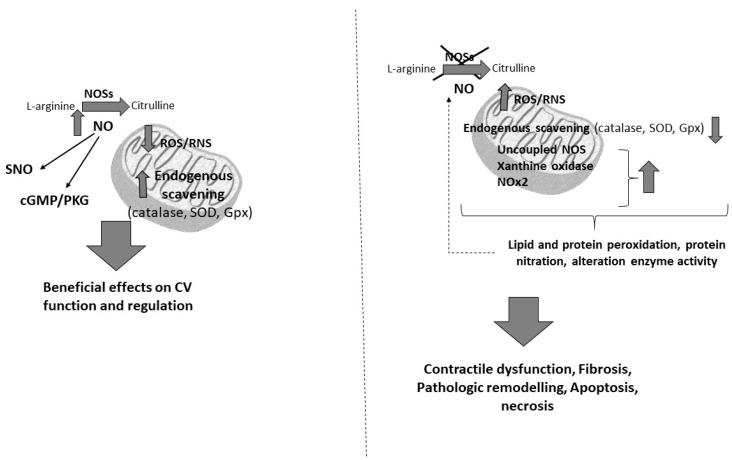
Mechanism action of reactive oxygen species (ROS). (RNS, Radical Nitrogen Species; CV, cardiovascular).

As aforementioned, oxidative stress plays a major role in CVDs and cancer in elderly subjects (Figure 1). Several studies have shown that cardiac tolerance to oxidative stress is reduced with ageing because of the lower concentrations of antioxidant enzymes (i.e., glutathione peroxidase and superoxide dismutase), thus favouring the development of CV alterations [12]. Moreover, there is a dramatic age-dependent increase in cancer risk, and high oxidative stress during the lifespan may be responsible for this phenomenon [11,12,24]. In fact, there is an accumulation of ROS-induced DNA damage with age, confirmed by the progressive and statistically significant enhancement in the levels of 7,8-dihydro-8-oxo-2′-deoxyguanosine (8oxodG) observed with ageing [25]. On these grounds, chronic inflammation and oxidative stress should be considered high risk factors for cancer, especially in elderly people [2,24]. In particular, the role of ROS is fundamental in the redox signalling pathways that are involved in different intracellular responses [26]. ROS play a dual role (physiologic or pathologic) depending on their type and concentration, and the site and time of production. In low physiological conditions, the ROS are involved in physiological processes, including excitation–contraction coupling (ECC), post-transcriptional modification protein (e.g., S-nitrosylation), cell differentiation and proliferation. In pathological conditions, e.g., ischemia/reperfusion injuries, concentrations are elevated, with effects on CV function (Figure 2). The production of ROS by redox cycling in cardiomyocytes induces mitochondrial dysregulation, lipid peroxidation, DNA damage, and protein carbonylation. High concentrations of ROS reduce the activity of endogenous antioxidants (e.g., glutathione peroxidase, catalase and SOD). The deleterious effects of ROS are augmented by DOXO, since it directly reduces the activity of antioxidant enzymes with a consequent increase in oxidative stress [27,28].

Several clinical approaches have evaluated the action of antioxidant therapies. Pharmacological approaches have different mechanisms: the inhibition of oxidative stress producers (i.e., xanthine oxidase and nitric oxide (NO) synthase (NOS) uncoupling), the improvement of endogenous antioxidant capacity with different antioxidants (i.e., N-Acetyl-L-Cysteine (NAC), vitamins A, C, and E), or drugs with anti-inflammatory and antioxidant properties (i.e., statins). Several clinical trials have shown that antioxidant pharmacological approaches are theoretically correct, but their results have not been very promising, until now. Future studies, new pharmacological formulations and more patient cohorts will allow for clearer results in order to identify more targeted therapies against cardiotoxicity (CTX) [26].

## 3. Metabolic Syndrome, Cancer and Cardiovascular Diseases

MetS is defined as a cluster of metabolic disorders that are associated with the increased risk of type 2 diabetes mellitus (DM2T) and CV events. These alterations include central obesity, high blood pressure, dyslipidaemia (raised triglycerides and/or lowered high-density lipoprotein cholesterol) and increased fasting glucose. The presence of at least three of these alterations allows for the qualification of a subject with MetS [29,30,31]. Its global prevalence has been estimated to be about one quarter of the world’s population [30]. Although its pathogenesis remains unclear, the predominant underlying condition with a key role in the molecular pathogenesis of these disorders is represented by insulin resistance. Interestingly, insulin resistance has been identified as a possible factor linking metabolic syndrome and cancer. In particular, adipose tissue dysfunction plays an important role by inducing insulin resistance, chronic inflammation, and changes in adipokines serum levels (including leptin and adiponectin), as well as sex steroids, and thereby promoting cell proliferation and survival, as well as invasive growth, metastasis, and angiogenesis [32,33]. In more recent years, a direct association between the development of diabetes and several different cancers (i.e., pancreas, breast, endometrium, colorectal [33,34,35], cutaneous and uveal melanoma [36]) has been demonstrated. Alterations in insulin and insulin-like growth factor type 1 (IGF-1) signalling pathways have been identified as the main drivers that lead to the development of both diabetes and cancer. From a pathophysiological standpoint, in the regulation of the carcinogenic process in patients with diabetes, specific alterations in both microRNAs and long non-coding RNAs affecting insulin signalling have been described [33]. In the *Vasterbotten* intervention project, it was clearly shown that, in women, the risk of developing cancer was higher in subjects with elevated fasting glucose level, and in both sexes, there was a significant increase in pancreatic, endometrial and urinary tract cancer, as well as melanoma [37]. Another possible pathophysiological mechanism proposed to explain the association between hyperglycaemia and cancer is the increased level of reactive oxygen species (ROS) produced in this circumstance [38]. Furthermore, metformin, an oral antidiabetic drug, has been shown to possess numerous pleiotropic effects and can have a role in anticancer treatment. Indeed, metformin can interfere with cancer metabolism by interacting not only with the pathways of adenosine monophosphate kinase and the mammalian target of rapamycin, but also with insulin itself and IGF-1 [39].

Additionally, lipid serum concentrations also seem to have a role in the development of cancer, and a significant positive association between colorectal cancer and higher levels of triglycerides has been shown in Europe [40]. Interestingly, a different correlation between lipids, sex and the incidence of colorectal cancer has been proven. Regarding colorectal cancer, its increased incidence has been associated with higher levels of triglycerides in adult males and elevated levels of total cholesterol in females [40].

Furthermore, subjects with MetS commonly manifest both a prothrombotic and a proinflammatory state. Chronic systemic low-grade inflammation is related to a higher risk of incident cancer, as suggested by the results of previous prospective cohort studies which found an increased risk of incident cancer related to higher C-reactive protein levels [41]. On the other hand, chemotherapy can induce alterations in the metabolism too, with an increase in cardiovascular risk. Antineoplastic agents can alter metabolic homeostasis, leading to MetS and its components.

Indeed, it is already well known that many anticancer treatments (such as some tyrosine-kinase inhibitors, platinum-based antineoplastics, and mitogen-activated protein kinase inhibitors [42] and vascular endothelial growth factor inhibitors [43]) can induce arterial hypertension.

Moreover, some antineoplastic treatments can directly or indirectly affect hormonal pathways, leading not only to hypogonadism, but also to dyslipidaemia and insulin resistance. For example, the low-testosterone levels that typically characterize patients treated with androgen deprivation therapy (ADT) for prostate cancer can be linked to a higher incidence of MetS in these subjects [44]. It has also been reported that ADT can impair the metabolism of both lipids and glucose [45], leading to an increase in cardiovascular diseases and MetS. Further studies are needed to fully understand the possible connection between metabolic impairment and ADT.

Childhood cancer survivors also have an increased risk of developing MetS as a direct consequence of antineoplastic therapies [46]. Brain cancer in children often requires treatment with radiotherapy (RT) and/or surgery that can damage the hypothalamic–pituitary axis, inducing MetS as a consequence of multihormonal deficiency. On the other hand, some chemotherapeutic agents can directly induce hypercholesterolemia, such as platinum-based antineoplastics [47], or hyperglycaemia, insulin resistance and diabetes, such as anthracyclines (ANT) [48] and immunotherapy [49]. Some antineoplastic treatments could even adversely affect the lipid metabolism years after the end of all anticancer therapies, leading to increased concentrations of triglycerides, total cholesterol and low-density lipoproteins [50].

Finally, the presence of MetS could represent a major risk factor for the development of cardiotoxicity [51]. As already mentioned above, subjects with MetS have a greater risk of cardiovascular disease. Whether the presence of MetS increases the susceptibility to chemotherapy-induced cardiotoxicity is a matter of debate. In this regard, it has been demonstrated that some of the features that define MetS have been associated with an increased risk of cardiotoxicity due to ANT and sequential ANT and trastuzumab in patients with breast cancer [52]. Furthermore, obese patients with cancer are characterised by a high rate of recrudescence or recurrence [53], leading to a poorer prognosis [54]. This could be related at least in part to an insufficient dose adjustment of antineoplastic drugs [55]. In order to improve the chemotherapy’s efficacy, antineoplastic drugs should be administered at higher dosages, but this could increase the risk of adverse reactions. Accordingly, cardiotoxicity is often dose-dependent and higher anticancer drug doses might increase the incidence of heart damages, as happens for ANT [51].

## 4. Hypercholesterolemia, Cancer and Cardiovascular Diseases

Oxidative stress, as the cause or consequence of impaired mitochondrial function, plays an important role in carcinogenesis, ageing, hypercholesterolemia and atherosclerosis [56,57]. According to the “*oxidative theory*” of atherosclerosis, [58,59] hypercholesterolemia (especially elevated low-density lipoprotein cholesterol), is a major risk factor for the development of atherosclerosis and subsequent ischemic heart disease [60]. Hypercholesterolemia is also associated with increased cardiac oxidative stress by mechanisms that are not entirely clear [61] (Figure 1).

Animal studies showed an increased superoxide production in the hearts of hypercholesterolemic animals, especially increased NADPH oxidase (NOXs) activity in cholesterol-fed Wistar rats and apoB100 transgenic mice [62]. In other studies, a link between NOX4 and miR-25 was found. NOX4 was identified as a direct target of miR-25, suggesting that decreased miR-25 allowed the upregulation of NOX4, which contributed to an increased ROS production in the hypercholesterolemic heart [63]. Hypercholesterolemia could also increase oxidative stress through the reduction of endogenous antioxidant capacity [64] and may directly affect the heart causing contractile dysfunction, aggravated ischemia/reperfusion injury and the diminished adaptation to ischemic stress [65,66,67,68,69]. In fact, cholesterol can have a direct effect on myocardial contractile function leading to impaired diastolic, and in some cases, also systolic function. In a cell culture model, an elevation of membrane cholesterol content in ventricular cardiomyocytes resulted in decreased cytosolic calcium levels and impaired cardiac myocyte contractility [70]. Hypercholesterolemia-induced cardiac dysfunction was further confirmed by echocardiography in humans [71]. Both the oxidation and nitrosylation of contractile proteins (tropomyosin and actin) have been hypothesised [72].

Hypercholesterolemia can increase the risk of cancer, especially breast cancer. In fact, in mammary tumours, hypercholesterolemia is associated with the increased expression of cyclin D1, a marker associated with tumour formation. The increased availability of high-density lipoproteins (HDL) may increase the oestradiol access to the cancer tissue [73]. The binding of HDL to scavenger receptor type BI activates Ras in a Protein kinase C (PKC)-independent manner, with the subsequent induction of the mitogen-activated protein kinase (MAPK) signalling cascade. In addition, oxysterol 27-hydroxycholesterol (27HC), synthesised from cholesterol by CYP27A1, can contribute to the progression of cancer. Actually, 27HC is a locally modulated, non-aromatised oestrogen receptor (ER) ligand that promotes ER positive breast cancer growth [74].

Importantly, some anticancer drugs can worsen dyslipidaemia. For example, the higher incidence of dyslipidaemia was found in the patients treated with nilotinib and other tyrosine kinase inhibitors [75].

Chemotherapy and radiotherapy contribute to increased oxidative stress. Oxidative stress remains the most probable mechanism for doxorubicin (DOXO)-induced cardiotoxic effects, mediated by the production of an iron complex and the subsequent generation of free radicals [76]. Moreover, methotrexate, bleomycin, cisplatin can cause cardiotoxicity, increasing oxidative stress. In experimental models, the potential role of ozone therapy in the management of chemotherapy-induced toxicity was investigated and it was associated with a decreased chemotherapy-induced toxicity [77].

In fact, it is known that chemotherapy and radiotherapy can cause cardiovascular complications and the risk of cardiotoxicity increases in the presence of cardiovascular risk factors. Patients with breast cancer and a combination of two or three comorbidities (hypercholesterolemia, obesity, diabetes) had an increased incidence of symptomatic cancer treatment-related cardiotoxicity [78,79].

Lipid metabolism is also associated with sex hormones. Premenopausal and postmenopausal women have different statuses of lipid metabolism, and dyslipidaemia is more common in postmenopausal women. In addition, cardiovascular diseases, chemotherapy-induced cardiotoxicity and cancer are more common in post-menopausal women [80]. Sexual hormone deficiency in postmenopausal women has been associated with metabolic changes, oxidative stress and subclinical inflammation [81]. Long-term hormonal depletion augmented the oxidative damage in the serum and peripheral tissues as well as increased the serum total cholesterol, tumour necrosis factor-α (TNF-α) and IL-6 levels [82].

Since ageing, cancer and hypercholesterolemia are associated with oxidative stress, the modulation of oxidative stress in elderly oncologic patients with hypercholesterolemia could be a rational approach. Considering the increased survival rate of cancer patients, cholesterol optimization should be considered beneficial in oncological patients, to prevent cardiovascular complication. Targeting oxidative stress may be a promising therapeutic strategy to reduce atherogenesis in patients with hypercholesterolemia and cancer [2,7].

The modulation of oxidative/nitrosative stress in hypercholesterolemia could be approached by at least three different ways: cholesterol-lowering therapies (statins, fenofibrates, ezetimibe) should be effective in the attenuation of oxidative/nitrative stress because they eliminate the cholesterol trigger effect; cholesterol-lowering drugs can have antioxidant properties; the support or induction of endogenous enzymatic antioxidant systems or the inhibition of the prooxidant enzymes [83,84].

## 5. Obesity, Cancer and Cardiovascular Diseases

Obesity is a major global health issue, not only for the relevant impact that this condition has on cardiovascular diseases, but also for the strict connection that exists between obesity and cancer. A recent publication underlines how obesity is considered a risk factor of different cancer types, including colorectal, post-menopausal breast, liver, endometrial, oesophageal, kidney, gastric, gallbladder, pancreatic, ovarian, thyroid, and multiple myeloma [85,86]. Tumour development and growth is known to be enhanced by the complex interaction between the tumour itself and multiple cells, mediators and other components [87,88]. In particular, adipocytes and macrophages promote inflammation, affect cancer cell metabolism, and facilitate tumour spreading, supporting its progression [89,90].

Different types of adipose tissue coexist in the human body, each with different functions and locations. Brown and beige adipose tissue are responsible for thermoregulation. Conversely, white adipose tissue is considered the gatherer of energy storage through triacylglycerides, stocked in visceral adipose tissue [91]. Furthermore, thanks to a higher concentration of mitochondria, visceral adipose tissue is metabolically more productive, greatly contributing to plasma-free fatty acid levels.

White adipose tissue has shown a strong correlation with cancer risk [92], mainly increasing the inflammation state by three main mechanisms: the secretion of inflammatory factors, enhanced tissue inflammation, and adipose tissue remodelling [93]. In addition, visceral fat has shown to be predictive of poor survival and treatment response in different oncologic setting [94,95,96].

Adipose tissue is also responsible for the production of inflammatory factors, particularly cytokines IL-6, IL-8 and tumour necrosis factor-alpha (TNF-α) and infiltrating cells, like macrophages, that can promote the event progression involved in the development and progression of cancer [97,98].

The effect of adipose tissue on cancer has been proven in different in vitro and in vivo studies [99,100,101,102]. Several studies underlined this strict crosstalk, particularly between obesity and breast cancer. Tumour-associated adipocytes act as an endocrine organ that induces: *(I)* tumour growth, producing a high concentration of tumour-promoting hormones, such as leptin and oestrogen and lowering the levels of tumour suppressor hormones, such as adiponectin, *(II)* enhanced tumour invasive capacity, by promoting local chronic inflammation through IL-6 secretion and *(III)* the establishment of a strict metabolic interaction based on the direct transfer of triglyceride-enhancing β-oxidation process and metastatic expansion [103,104,105]. Furthermore, in patients affected by breast cancer, obesity has been proven to be associated with a poor outcome in patients treated with DOXO [106]. Whether this is linked to a higher cardiovascular risk, or due to lower socioeconomic status and/or unfavourable genetic background, is still debated [107]. In an elegant study, Burridge and colleagues reported that human-induced pluripotent stem cell-derived cardiomyocytes (hiPSC-CMs) from breast cancer patients who had developed doxorubicin cardiotoxicity (CTX) were consistently more sensitive to doxorubicin toxicity than the hiPSC-CMs from subjects who did not develop CTX, demonstrating that this cellular model is an interesting approach for the identification of the genetic basis and molecular mechanisms of CTX [27]. Similarly, in another very recent study, organoids have been proposed as a model that can recapitulate hypoxia-enhanced doxorubicin cardiotoxicity [108].

Conceivably, besides the increased risk of developing cancer, another relevant issue linked to obesity and overweight is the susceptibility to chemotherapy-induced cardiac disease.

In a metanalysis including 15 studies and 8745 patients, Guenancia and colleagues showed that overweight and obesity are significantly associated with the risk of cardiotoxicity, whether the patients are treated with ANT alone or sequential ANT and trastuzumab (OR:1.38; 95% IC, 1.06–1.80) [52]. This was confirmed by a recent large-scale prospective study including 929 patients undergoing ANT and/or trastuzumab, in which obesity was associated with a three-fold increased risk of developing cardiotoxicity, regardless other predictors of cardiotoxicity [109].

The mechanisms that increase the risk of developing cardiotoxicity among obese patients are yet to be elucidated and may be determined by different factors. One possible cause can be found in the lower clearance rate of some anticancer treatment in patients with higher percentage of ideal body weight [110]. The development of obesity in male rats fed with a high-fat diet seems to sensitize ANT-induced cardiotoxicity by affecting the cardiomyocyte metabolism in multiple ways, mainly resulting in increased oxyradical stress, reduced cardiac fatty acid oxidation, with concomitant lower cardiac ATP levels, and decreasing plasma adiponectin levels [111,112].

Furthermore, in vivo models showed that adiponectin-KO mice treated with DOXO were keener to develop left ventricular dysfunction compared to wild type. In the same model, exogenous adiponectin improved DOXO-induced left ventricular dysfunction in both wild type and adiponectin KO mice [113].

The pivotal effect of the adiponectin system has been showed in a murine model injected with DOXO. Pre-treatment with metformin, which increases the endogenous level of adiponectin and its receptor, attenuates the detrimental effects of anticancer therapy increasing cell viability, reducing the activation of caspases and the fragmentation of genetic material, and restoring the antioxidant activity [114].

Finally, as the survival rate of patients diagnosed with cancer has significantly improved over the years, the resulting cancer survivor population showed an increased risk of morbidity and mortality for cardiovascular diseases compared to the non-cancer population, mainly occurring as the late effects of previous cancer treatment (e.g., ANT chemotherapy and/or chest-directed radiotherapy). However, the impact of modifiable cardiovascular risk factors such as hypertension, diabetes, dyslipidaemia and obesity seem to exert a considerable impact on the risk for a severe, life-threatening cardiac event, independently from cancer therapy-related risk [115,116].

Reduced physical activity, sarcopenia, and endocrine disorders including hypothalamic dysfunction, hypothyroidism and hypogonadism play a crucial role in the development of overweight and central obesity in cancer survivors [117]. The use of corticosteroids (which represent the first-line treatment for graft versus host disease, and are widely employed in hematologic patients treated with hematopoietic stem cell transplantation) contributes to the production of alterations in body mass and composition in these patients [118]. ADT, which is also the backbone of anticancer treatment in elderly patients with locally advanced or metastatic prostate cancer, may cause a range of metabolic alterations, including obesity [118].

Therefore, the assessment and appropriate management of cardiovascular risk factors, including obesity, in cancer survivors, may substantially reduce the incidence of premature cardiac disease.

## 6. Hypertension, Cancer and Cardiovascular Disease

Hypertension in cancer patients has a prevalence comparable to the general population [119]. In a registry including 17,712 patients, hypertension resulted as the most frequent comorbidity, showing a prevalence of 38% [120]. Moreover, some of the risk factors for both malignancies and hypertension are shared (i.e., obesity) [121,122].

Cancer patients affected by hypertension show a higher risk of developing cardiotoxicity secondary to antineoplastic therapy, of which the left ventricular dysfunction is the most concerning condition [120]. In the American Society of Clinical Oncology guidelines, arterial hypertension, dyslipidaemia, coronary artery disease and age > 65 years are considered risk factors for cardiotoxicity induced by antiblastic therapy. Both animal and human studies highlighted the weight of arterial hypertension in inducing cardiotoxicity, particularly in ANT-based therapy protocols [123,124,125,126].

Hypertension and cancer share a wide range of pathophysiological pathways: the increased activity of Angiotensin II (ANGII), vasopressin and catecholamines, alterations in calcium homeostasis, inflammation, high levels of ROS, disturbances in the levels and activity of growth factors, mainly angiogenic; all these factors could interplay in a vicious circle that reciprocally links cancer and hypertension [122].

The role of ROS in malignancies is well known: the neoplastic status enhances ROS production, and oncogenic pathways are able to set ROS homeostasis to a higher level, thus leading to a maintaining of the cancer cell phenotype [2,7,127]. On the other hand, under physiological conditions, ROS modulate cellular processes, such as differentiation, proliferation, apoptosis, cell cycles, migration [128,129,130] and regulate endothelial function and vascular tone in the vascular system. Unbalanced ROS generation causes oxidative stress, decreases NO production and is able to impair antioxidant defences in the cardiovascular system. In such a context, endothelial dysfunction (reduced vasodilation, increased vasoconstriction and loss of endothelial integrity) leads to the development of arterial hypertension. Indeed, ROS play an important role both in the pathophysiology of cancer and of hypertension [128,129,130] (Figure 1).

Furthermore, hypertension is a frequent adverse effect of anticancer therapies [131]. In the setting of anticancer therapies, about 30% of patients develop hypertension in the course of the disease’s natural history. It is worth nothing that hypertension showed a higher prevalence in patients receiving chemotherapy [123].

Several agents may induce or worsen previously well controlled hypertension. Vascular endothelial growing factor inhibitors, tyrosine kinase inhibitors, cisplatin derivatives, proteasome inhibitors, corticosteroids, alkylating agents, interferon-alpha, the mammalian target of rapamycin inhibitors, taxanes, vinca rosea alkaloids and gemcitabine, immunosuppressive agents and nonsteroidal anti-inflammatory drugs are all therapies potentially able to cause hypertension, mainly determining, through different pathways, endothelial dysfunction [123].

Besides modulating blood pressure and myocardial remodelling, the renin–angiotensin–aldosterone system (RAAS) plays a central role in modulating ROS production [129,130,131,132]. Enalapril, administered for cardioprotection during ANT treatment, was able to reduce the rate of the development of symptomatic left ventricular (LV) dysfunction [133]. Similarly, ANGII-receptor blockers candesartan, telmisartan, and valsartan exhibited the ability to modulate the cardiotoxicity induced by ANTs [134,135], while the positive effect of β- blockade in cancer treated patients was also contributed by the decrease in oxidative stress and myocardial calcium overload [136]. However, only the newest beta blockers (e.g., carvedilol and nebivolol) proved to be effective as a cardioprotective strategy in ANT-induced LV dysfunction. This effect has been attributed to their antioxidant properties rather than to their beta-AR blocking action [137]. On the contrary, bisoprolol and metoprolol were not demonstrated to be effective as cardioprotective agents [138], implying that the β1 blockade alone, without a specific effect in reducing oxidative stress, is not adequate to guarantee successful cardioprotection [139,140].

## 7. Diabetes and in Cancer and Cardiovascular Diseases Cardiotoxicity

Diabetes mellitus and cancer are major causes of morbidity and mortality worldwide [141]. Moreover, epidemiological studies have demonstrated that there is a strong link between certain cancers and DM2T; thus, it is not uncommon to find cancer and diabetes in the same patients. Diabetes mellitus is an established cardiovascular risk factor, and can also be responsible for a specific cardiomyopathy, known as diabetic cardiomyopathy [142,143]. Cancer patients with long standing diabetic cardiomyopathy may constitute a challenge when undergoing chemotherapy. Understanding the possible interactions with diabetes is of paramount importance in the complex management of these patients, because of the known cardiotoxic effects of some anticancer treatments. Diabetic cardiomyopathy is a form of heart disease secondary to insulin-resistance and hyperglycaemia, progressing from subclinical cardiac abnormalities to severe diastolic heart failure with preserved ejection fraction (HFpEF) and eventually to systolic dysfunction characterised by heart failure with reduced ejection fraction (HFrEF) [144,145]. Pathophysiological changes, including impaired cardiomyocyte autophagy, increased cardiomyocyte death, inappropriate RAAS activation, oxidative stress and maladaptive immune responses, result in fibrosis, cardiac remodelling and substantial cardiac stiffness/diastolic dysfunction. In particular, oxidative stress, due to the enhanced production of ROS in the heart driven by hyperglycaemia, is believed to be involved in the pathogenesis of this condition [146]. The mechanisms that generate ROS in the diabetic heart include mitochondrial electron leakage, the activity of ROS-generating enzymes such as NADPH oxidase, xanthine oxidase and 12/15 lipoxygenase, the uncoupling of nitric oxide (NO) synthase (NOS), the accumulation of advanced glycation end-products (AGEs) and the activation of PKC. A common upstream pathway is the interaction of AGEs with their receptor-AGE (RAGE), which further promotes ROS synthesis [146,147]. It has been extensively demonstrated that DOXO induces myocardial damage, mainly through oxidative stress, due to the enhanced production of ROS [8,148]. Thus, one concern about DOXO therapy is its toxicity in patients who are more prone to toxic side effects, particularly in patients with comorbidities such as diabetes mellitus. Indeed, patients with breast cancer and diabetes are at an increased risk of chemotherapy-related toxicities compared to non-diabetic patients [149].

Currently, the molecular mechanisms underlying the DOXO-induced cardiotoxicity in diabetic hearts are largely unknown. In a study investigating acute DOXO cardiotoxicity in streptozotocin-induced diabetes (STZ-DM) and non-DM rats, the elevated heart accumulation of DOXO was found in diabetic rats, while plasma and renal clearance were reduced. In streptozotocin-induced diabetes (STZ-DM) rats, an increase in creatine phosphokinase, used as a marker of DOXO cardiotoxicity, was found [150]. Moreover, STZ-DM rats showed an enhanced reduction of heart rate compared to non-diabetic rats, probably related to calcium dysregulation. Both diabetes and DOXO, in fact, are known to induce myocyte calcium overload. Therefore, it is possible that the synergistic action of DOXO and diabetes leads to excessive myocyte calcium overload, combined with the oxidative stress induced by both diabetes and DOXO.

More recently, a panel of regulatory genes associated with cardiac remodelling, inflammatory response and oxidative stress in the setting of DOXO cardiotoxicity in diabetic hearts were investigated. In particular, S100 calcium binding protein A8 (S100A8) and A9 (S100A9), members of the S100 family implicated in inflammatory response and immune disease, were found to have a specific pattern in DOXO cardiomyopathy in the diabetic heart [151]. The authors found that DOXO upregulated S100A8/S100A9 expression in the hearts of diabetic mice, promoting nuclear factor kappa-light-chain-enhancer of activated B cell (NF-κB) activation through the p38 MAPK signalling pathway and inducing cardiac inflammation as demonstrated by the elevation of the cardiac IL-6 level [151]. For this reason, the authors propose the pharmacological blockade of S100A8/A9 as a possible promising cardioprotective strategy in cancer patients with a history of diabetes.

Due to the cardiac unfavourable effects of both cancer therapies and diabetes mellitus, identifying the best antidiabetic treatment in these patients is of paramount importance. The cardioprotective effect of both metformin and sitagliptin was demonstrated in a model of DOXO-induced cardiotoxicity in non-diabetic rats [152,153]. These drugs possess antioxidant properties and also have anti-inflammatory and antiapoptotic effects, as demonstrated by the decreased expression of the proinflammatory cyclooxygenase-2 and inducible NOS enzymes, as well as of the proapoptotic executioner caspase-3 enzyme. Thus, metformin and/or sitagliptin may be suggested to diabetic patients treated with doxorubicin. In addition, more recently, sodium-glucose co-transporter 2 (SGLT2) inhibitors, a new class of antidiabetic agents that improve glycaemic control, were shown to reduce the risk of heart failure-associated hospitalization and mortality in patients with diabetes. Interestingly, it was demonstrated that SGLT2 inhibitors attenuated DOXO-induced cardiotoxicity in mice [154]. This protective effect was mediated by elevated beta-hydroxybutyrate levels, which reduced ROS production and improved mitochondrial dysfunction in cardiomyocytes. Therefore, even if more data are needed, these preliminary findings encourage the possible use of SGLT2 inhibitors as a new strategy to prevent HF in patients receiving DOXO.

## 8. Conclusions

Cancer patients often present with cardiovascular comorbidities, and patients with cardiac risk factors are often at risk of developing cancer, too. Indeed, cancer and CVDs share many risk factors [155], including smoking, ageing and MetS and its components (hypertension, obesity, hypercholesterolemia). Alterations in ROS may have a pivotal role connecting malignancies and CVDs. Indeed, both cancer and many CVDs are characterised by chronic inflammation and augmented ROS production [22,156]. In addition, some anticancer treatments can induce cardiotoxicity, by both directly affecting cardiomyocytes as well as augmenting ROS generation, ultimately leading to an increased risk of developing, among others, arterial hypertension and obesity [45,157,158]. Patients treated with antineoplastic therapies can have a sometimes significant incidence of cardiac events, also due to ageing, as well as MetS and CV risk factors, comorbidities characterised by imbalance in redox and inflammatory homeostasis [2,159]. On the other hand, the presence of CVDs themselves may trigger carcinogenesis by inducing chronic inflammation and promoting cancer cell development [22,155] (Figure 1).

Unfortunately, oncologists have traditionally not paid much attention to the heart, perhaps due to the fact that the cardiomyocytes typically do not turn into malignant cells. Although atrial fibrillation and other arrhythmias as well as myocardial infarction are further consequences (beyond HF) that may be caused by antineoplastic therapies, cardiology and oncology have not developed as interconnected medical disciplines, the surge of cardio-oncology being a recent phenomenon [23].

Further research investigating the link between ageing, MetS, CVDs and cancer may help to better characterize the mechanisms and novel approaches for the prevention of antineoplastic drug cardiotoxicity.

## Figures and Tables

**Table 1:** Cardiovascular risks associated with cancer therapies [5,6,7,8,9,10].

Treatments	Toxicities
Anthracyclines, HER2-targeting drugs, VEGF/multitargeted RTK inhibitors, proteasome inhibitors, radiation therapy	Left ventricular dysfunction
Fluoropyrimidines, VEGF inhibitors, radiation therapy	Myocardial ischemia
Immune checkpoint inhibitors, cyclophosphamide	Myocarditis
Ibrutinib	Atrial fibrillation
Arsenic trioxide, vantedanib, androgen deprivation therapy	QT prolongation
radiation therapy	Valvular heart disease
Immune checkpoint inhibitors, cyclophosphamide	Pericarditis
VEGF inhibitors	Hypertension
Nilotinib, ponatinib	Peripheral artery disease
Cisplatin, nilotinib, ponatinib, thalidomide and lenalidomide, VEGF inhibitors, proteasome inhibitors, aromatase inhibitors	Vascular thrombosis
Dasatinib, cyclophosphamide	Pulmonary arterial hypertension

VEGF, Vascular endothelial growth factor; RTK, Receptor Tyrosine Kinases; HER2, human epidermal growth factor receptor 2.

## Abbreviations

27HC	27-hydroxycholesterol
8oxodG	7,8-dihydro-8-oxo-2′-deoxyguanosine
ADT	androgen deprivation therapy
AGEs	advanced glycation end-products
ANGII	angiotensin II
ANT	anthracyclines
CV	cardiovascular
CVD	cardiovascular diseases
DM2T	type 2 diabetes mellitus
DOXO	doxorubicin
ER	oestrogen receptor
HDL	high-density lipoproteins
HF	heart failure
HfpEF	HF with preserved ejection fraction
IGF-1	Insulin-like growth factor 1
IL	interleukin
LV	left ventricular
MAPK	mitogen-activated protein kinase
MetS	metabolic syndrome
NF-κB	nuclear factor kappa-light-chain-enhancer of activated B cells
NO	nitric oxide
NOS	NO synthase
NOXs	NADPH oxidase
PKC	protein kinase C
RAAS	renin–angiotensin–aldosterone system
RAGE	receptor-AGE
ROS	reactive oxygen species
RT	radiotherapy
S100A8 and S100A9S100	calcium binding protein A8 and S9
SGLT2	sodium-glucose co-transporter 2
STZ-DM	streptozotocin-induced diabetes
TNF-α	tumour necrosis factor-alpha
